# Short-term exposure to ambient fine particulate pollution aggravates ventilator-associated pneumonia in pediatric intensive care patients undergoing cardiovascular surgeries

**DOI:** 10.1186/s12940-023-00991-y

**Published:** 2023-04-26

**Authors:** Zhaomei Cui, Yingying Ma, Yuanyuan Yu, Na Li, Jun Wang, Anbiao Wang, Qi Tan

**Affiliations:** 1 Intensive Care Unit (ICU), Department of Cardiac Surgery, Shandong Provincial Hospital Affiliated to Shandong First Medical University, No. 9677 Jingshi Road, Jinan, 250021 China; 2 Medical Engineering Department, Shandong Provincial Hospital Affiliated to Shandong First Medical University, Jinan, China; 3 Data Science Institute, Shandong University, Jinan, Shandong China; 4 Department of Gynecology, Shandong Provincial Hospital Affiliated to Shandong First Medical University, Jinan, Shandong China; 5 Department of Pharmacy, Shandong Provincial Hospital Affiliated to Shandong First Medical University, Jinan, China

**Keywords:** Particulate matter (PM_2.5_), Intensive care unit (ICU), Ventilator-associated pneumonia (VAP), Pediatric population, Cardiovascular surgery

## Abstract

**Background:**

Ambient air pollutants can be hazardous to human health, especially for vulnerable children. The impact of ambient air pollutant exposure before and during intensive care unit (ICU) stays on the development of ventilator-associated pneumonia (VAP) in critically ill children has not been established. We aimed to determine the correlations between short-term exposures to ambient fine particulate matter (PM_2.5_) and VAP in pediatric cardiac surgery patients in the ICU, and explore the effect of delayed exposure.

**Methods:**

The medical record of 1755 child patients requiring artificial ventilation in the ICU between December 2013 to December 2020, were analyzed. The daily average concentrations of particulate matters (PM_2.5_ and PM_10_), sulfur dioxide (SO_2_), and ozone (O_3_) were calculated from public data. Interactions between these pollutants and VAP were simulated with the distributed lag non-linear model.

**Results:**

Three hundred forty-eight cases (19.829%) of VAP were identified in this study, while the average concentrations of PM_2.5_, PM_10_, O_3_ and SO_2_ were 58, 118, 98 and 26 μg/m^3^, respectively. Exposure to increased levels of PM_2.5_ two days prior (lag 2-day) to VAP diagnosis is significantly correlated with an enhanced risk for VAP development. Even a slight increase of 10 μg/m^3^ in PM_2.5_ can translate to a 5.4% increase in VAP incidence (95% CI: 1.4%-9.5%) while the VAP incidence increased to 11.1% (95%CI: 4.5–19.5%) when PM_2.5_ concentration is well below the National Ambient Air Quality standard (NAAQS) of 50 μg/m^3^. The association was more pronounced in those aged below 3-months, with low body mass index or suffered from pulmonary arterial hypertension.

**Conclusion:**

Short-term PM_2.5_ exposure is a significant risk for development of VAP in pediatric patients. This risk is present even with PM_2.5_ levels below the NAAQS. Ambient PM_2.5_ may represent a previously unrecognized risk factor for pneumonia and the current environmental pollution standards need to be reevaluated to consider susceptible populations.

**Trial registration:**

The trial was registered with the National Clinical Trial Center: The correlation between ambient air pollution and the complications in ICU underwent cardiac surgery. Trial registration number: ChiCTR2000030507. Date of registration: March 5, 2020. URL of trial registry record: http://www.chictr.org.cn/index.aspx.

**Supplementary Information:**

The online version contains supplementary material available at 10.1186/s12940-023-00991-y.

## Introduction

Environmental factors such as air pollution are increasingly impacting human health around the world, contributing to about 22% of global death and disability according to the World Health Organization (WHO) [1]. Particulate matters in haze, especially those ≤ 10 µm (PM_10)_ and ≤ 2.5 µm (PM_2.5_), are of major concern, with PM_2.5_ being the primary air pollutant in China [2, 3]. The WHO has developed guidelines to help countries achieve safe levels of these particles incrementally; for example, the Interim Target 1 (IT-1) for 24-h mean PM_2.5_ is 75 μg/m^3^, with reduction to 25 μg/m^3^ by IT-4. The current level set by the China National Ambient Air Quality Standards (NAAQS) for PM_2.5_ is 75 μg/m^3^ (GB3095-2012), equivalent to the WHO IT-1 level, with the goal to reach 50 μg/m^3^ for the next stage. However, nearly 86% of the populations in China still experience extreme concentrations of PM_2.5_, well above the current standard [4]. Air population from ambient particulate matters is a significant health risk for children below 10 years of age. Systematic analysis of 204 countries and regions identified 808,694 global deaths in children up to 5-years-old (including 107,811 Chinese children) in 2017, mainly due to aggravation of respiratory diseases [5].

Short-term PM_2.5_ exposures have been correlated with mortality or respiratory system diseases in several studies of adults in well-monitored metropolitan areas [6]. However, children may have undeveloped respiratory and immune systems, and poor nutritional status, particularly in rural areas. Thus, more evidences are required for this population group to determine the effect of ambient PM_2.5_ exposure on health. Some supporting evidence showed that among children hospitalized for pneumonia, positive associations were seen with short-term daily exposure to ambient air pollution [7, 8]. Similarly, pediatric patient visits to the emergency department for respiratory symptoms were also increased in those with high levels of daily PM_2.5_ exposure [9]. To date, the NAAQS of China has yet to set air pollutant standards to protect “sensitive subgroups”. This may be due to insufficient data demonstrating direct causal effects between air pollution and health complications such as nosocomial pneumonia in intensive care unit (ICU) stays since most studies are correlative studies based on outpatient visits or death registration. Thus, more studies are required for specific subgroups (e.g., newborns and pediatric surgery patients) to evaluate the health impacts of acute pollutant exposure.

Nosocomial pneumonia, infection acquired during hospitalization, is prevalence in children, particularly those in pediatric and neonatal ICUs [10]. Mechanical ventilation can induce ventilator-associated pneumonia (VAP) 48 h after intubation, which increases length of hospitalization, broad-spectrum antibiotic usage and morbidity [11, 12]. Rates of pneumonia development are 6- to 20-folds higher in patients requiring mechanical ventilation than those who do not, and this subsequently results in the lengthening of mechanical ventilation hours by 10 to 20 times [13, 14]. VAP has been shown to significantly affect the composition of the lung microbiota and should be viewed as an abrupt and emergent disruptor of the complex homeostasis of the respiratory system [15]. The respiratory system is particularly vulnerable to fine particulate matters that can penetrate deeply into the lung epithelium. Atmospheric pollution, especially fine particulate matters, has been shown to activate macroautophagy and increase interleukin-18 levels in lung epithelial cells [16, 17]. Children are uniquely vulnerable to air pollution owing to their underdeveloped immune systems, high ventilation rates, and frequent infections with respiratory pathogens [18, 19]. Therefore, long-range studies with larger sample sizes will improve our understanding of the adverse influence of air pollutants on young ICU patients, and may contribute to development of early intervention strategies to improve perioperative management in the ICU.

To evaluate the effect of short-term PM_2.5_ exposures on VAP risk in children and subgroups, we used a time-series analysis method based on the distributed lag non-linear model (DLNM) to examine VAP incidence in a single cardiac center from 2013 through 2020. Both value and temporal lag dimensions were also evaluated.

## Materials and methods

### Trial design and oversight

This was a single-center, ambispective cohort trial that received approval from the Shandong Provincial Hospital Affiliated to Shandong First Medical University (approval number 2018–239). In addition, the study was registered with the National Clinical Trial Center (ChiCTR2000030507). The research was carried out in accordance with the STROBE statement. The information relevant to this cohort study was collected from electronic medical records or follow-up record sheets, after purging personal information of all the patients. The Ethics Committee waived the need for informed consent by participants whose data were obtained from medical records before February 28, 2020 (the date of ethics approval), while written informed consent was obtained from patients (or their legal representatives) who were enrolled in the study after that date. All authors affirmed that the data and analyses in the trial were accurate and complete, and that the trial was conducted in a manner consistent with the study protocol. The statistical analysis was performed by Cheeloo College of Medicine, Shandong University. The Shandong Centers for Disease Control and Prevention served as a neutral third party which supported data management and quality control.

### Study population

This study was conducted at the 14-bed tertiary ICU of Shandong Provincial Hospital Affiliated to Shandong First Medical University, with the biggest cardiac surgery center of Shandong province. To reduce surgical technique bias, we chose the fixed ward with an experienced medical team consisted of six professors, each with experience of 3000 solo operations.

Patients who underwent cardiovascular surgery and fulfilled the following criteria were included: (1) less than 6 years old; (2) long-term resident of Jinan city or cities with meteorological air quality index (AQI) within 10% of the Jinan index. The exclusion criteria were: (1) existing pneumonia up to seven days before surgery; (2) multiple organ failure before surgery; (3) administration of endotracheal intubation before surgery.

**Fig. 1:**
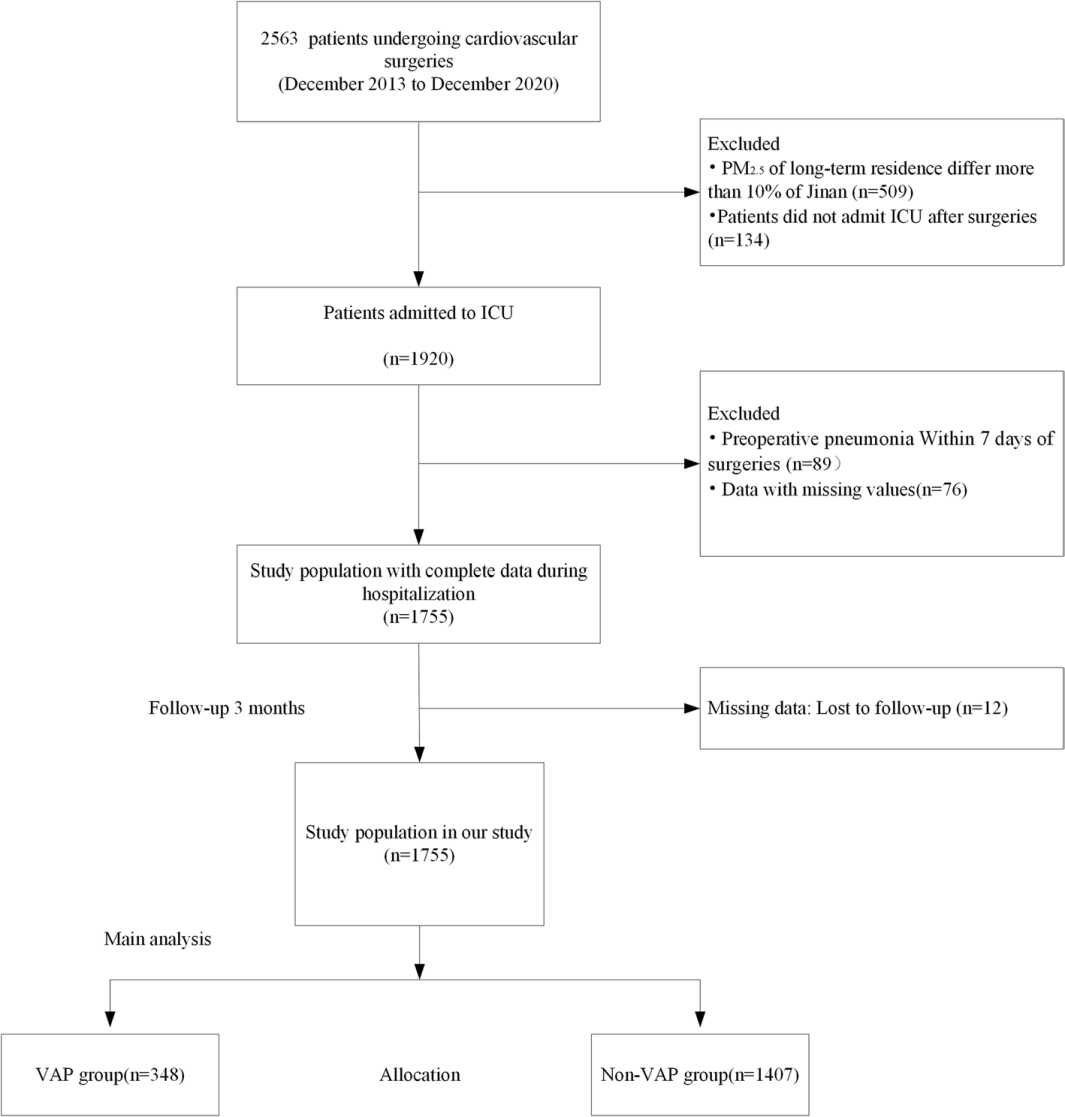
Schematic of patient selection strategy. Notes: ICU: Intensive care unit; VAP: ventilator-associated pneumonia

The medical records of 2563 patients, admitted from December 2013 through December 2020, were analyzed for all modes of admission to the ICU. Clinical data were obtained from the patient data management system, while personal information (pervious medical history and history of medication) was extracted from other medical records. After exclusion of patients with more than 10% difference in PM_2.5_ level of their residence compared to Jinan city (*n* = 509); patients who were not admit to ICU after surgery (*n* = 134); patient with preoperative pneumonia within seven days before surgery (*n* = 89); data with missing values (*n* = 76); a final 1755 patients were included in this study (Fig. 1).

Relevant demographic and clinical data were obtained for all selected patients. Ventilation duration is the total number of hours that the patient received invasive ventilation during the same ICU admission. ICU stay and postoperative hospital stay were collected by days. To protect patient confidentiality, all records were deidentified before analysis. Quality control and data management were performed by Shandong Centers for Disease Control and Prevention.

### Setting

Shandong Provincial Hospital is located in Jinan, the capital city of Shandong Province, China. Jinan was selected for our study because it ranks highly among polluted cities of China. Jinan is a northern inland city at 36° 40′ latitude and 116° 57′ east of Greenwich, with complex natural terrains and poor airflow and ventilation. Situated about 400 km south of Beijing, the city covers an area of 8227 km^2^, containing 12 administrative regions and a population of 9.32 million. The major industries that contribute to approximately 40% of Jinan’s gross domestic product (GDP) include manufacturing of steel, machineries, chemicals and textiles, light and construction industries, and food processing. The climate of Jinan is typically warm, temperate, with semi-humid monsoon seasons and an annual average temperature of 13.8 °C. The three winter months from December to February have mean temperatures around 0 °C.

### Ambient air pollution exposure assessment

#### Air pollution and meteorological conditions in Jinan

China first introduced monitoring of PM_2.5_ levels as a part of air quality indicators in 2012, and monitored PM_2.5_ concentrations in 113 cities from 2013, with data released to the public from November of the same year. Thus, we analyzed all air pollution records from January to December 2020. The following indices were provided by Jinan Environment Monitoring Center: daily average concentrations of fine particles (PM_2.5_), inhalable particles (PM_10_), and sulfur dioxide (SO_2_); 24-h average concentration of nitrogen dioxide (NO_2_); average concentration of ozone (O_3_); air quality index (AQI), maximum and minimum temperature (T_max_ and T_min_), humidity, wind force, and wind direction. The AQI was divided into 6 categories: hazardous (301–500); very unhealthy (201–300); unhealthy (151–200) unhealthy for sensitive groups (101–150); moderate (51–100); good (0–50). Daily temperature, relative humidity and wind speed data were provided by the China Meteorological Data Sharing Service System. Public holidays were obtained from public records.

#### Air pollution exposure in and around the ICU

Our ICU is installed with conventional centralized ventilation systems for air filtration and not equipped with laminar flow systems. PM_2.5_ levels were measured indoors (at the center of the ICU) and outdoors (10 m outside the ICU) from January to December of 2020 to correlate the indoor and outdoor air quality. Air sampling was performed with two portable testing equipment (Lvchi PM_2.5_ detector, made in China). Measurements were performed at 8:00AM and 2:00 PM with values recorded after the instruments were placed at their respective locations for half an hour, with the average of the two readings taken as the final value for the day. The instruments were calibrated every month to maintain functionality.

### Outcome

The study outcome is VAP, which is defined as nosocomial pneumonia that developed 48 h or more after patients were intubated for mechanical ventilation [10]. Two clinicians were required for VAP diagnosis and patients with unverified or missing data were excluded from the study (Fig Supp. S1).

### Missing data

The proportion of missing data in this study was low. Seventy-six patients were found to have missing data, specifically, a lack of relevant examination data, and we excluded these cases. For 12 patients who were lost to follow-up, missing counts were replaced with negative results.

### Statistical analysis

The sample size required for regression modeling can be estimated based on the principle of approximately 10 outcome events per variable that can affect the outcome [20]. In our case, approximately 20 variables have the potential to affect VAP in patients after cardiovascular surgeries (Suppl.). Thus, the minimum sample size required is approximately 200. Based on our hospitalization records, the incidence of VAP was 17.2% between 2011 and 2012. We expect the VAP incident in our current study to be 15–20%. Hence, the sample size should be 1200. Considering that larger sample sizes will allow more realistic interpretations, we decided to evaluate 1.4-folds more patients than the estimated samples size. Therefore, a final cohort of 1755 patients were included, and the database was locked on December 30, 2020.

For statistical analysis, categorical data were presented as proportions and evaluated with χ^2^ tests; continuous data were presented as mean and standard deviation (SD) or median with inter-quartile-range (IQR). Parametric data were evaluated with Student’s t-tests or one-way ANOVA, while non-parametric data were analyzed with equivalent non-parametric tests to compare the difference between VAP and non-VAP groups. Relationships between daily concentration of air pollutions and meteorological factors, and between ambient and indoor PM_2.5_ concentration were analyzed by Spearman rank correlation. We assume that the effect of air quality on VAP develops over time as the impacts on the respiratory system is likely to be lag and cumulative. Thus, we used distributed lag non-linear models (DLNMs) to identify any delayed effects and the relative risk (RR) of PM_2.5_ on VAP incidence. The DLNMs simulate potentially non-linear and delayed dependencies between air quality parameters and VAP [21]. The effect of mean daily pollutant exposure for up to seven lag days (days prior to VAP diagnosis) were evaluated, with “lag 0-day” denoted as the day of VAP diagnosis, and “lag7-day” as 7-days prior. The cumulative effect was also evaluated between VAP diagnosis day and 1-day prior (lag 01-day), 2-day prior (lag 02-day), etc.

DLNMs are a modeling tool that describes potentially non-linear and delayed dependencies [20]. The main advantage of this method is that it allows the model to include a detailed representation of the time-course of the exposure–outcome relationship, and can simultaneously control for exposures during the different time periods. In this study, the DLNM simulated the pollutants as main effectors but natural splines for air and dew point temperatures with 5 degrees of freedom (df) were included to control for potential confounding by meteorological variables using the following equation:

$$\mathrm{log}\left(E\left({y}_{t}\right)\right)=\alpha +s\left({x}_{t};{\varvec{\beta}}\right)+factor\left({DOW}_{t}\right)+ns\left(TMAX,df\right)+ns\left(TMIN,df\right)+ns\left(HY,df\right)$$

Where the subscript *t* refers to the day of the study; $E\left({y}_{t}\right)$ represents VAP incidence in the ICU; ${x}_{t}$ is the daily concentration of ambient fine particulate pollution, such as PM_2.5_; β represents the log-relative risk of VAP incidence per unit increase of ambient pollution; ns is the natural spline function. We included per year for calendar time, day of the week (DOW) and meteorological variables, such as maximum (T_MAX_) and minimum temperatures of the day (T_MIN_), and humidity (HY). Exposure–response curves were estimated for PM_2.5_ and VAP incidence by replacing linear terms for the three pollutants (PM_2.5_, SO_2_ and O_3_) with penalized splines for PM_2.5_.

Modeling was also performed for subgroups of patients based on gender (male and female); age (≤ 0.3 year, 0.3 to 0.8 year, 0.8 to 2 years, and 2 to 6 years); the season during which the surgery was conducted (warm and cold seasons); BMI (≤ 17 and > 17); risk adjustment for congenital heart surgery (RACHS) classification (≤ 2 and > 2); whether pulmonary hypertension (PH) was present [22]. Separate Z-tests were performed for each subgroup data to obtained subgroup-specific RR scores [23]. Two-sample tests were used to determine the statistical significance of the RR scores between the categories of each subgroup (eg, female vs male), taking into account the RR score and the standard error (SE). All statistical analyses were performed with the R software (version 4.2.2). Significance was defined as two-tailed *P* < 0.05.

## Results

The various demographic and clinical characteristics of the 1755 child patients are summarized in Table 1. 1280 (72.9%) patients were less than 1 years old while 89.86% patients belonged to the pediatric population (less than 3 years old). There were slightly more boys (53.3%) than girls (46.7%). The mean (± SD) BMI showed a remarkably low value of 15.003 ± 2.41. The median ventilation duration was 24 h (minimum 6 h, maximum 70 h), and 348 patients were diagnosed with VAP.

**Table 1:** Clinical characteristics of patients involved in the study

Among all cases (*n* = 1755)	
Age, y	0.8(0.3, 2)
0–1 years(No, proportion)	1280 (72.934%)
1–2 years (No, proportion)	168 (9.573%)
2–3 years (No, proportion)	129 (7.35%)
3–4 years (No, proportion)	69 (3.932%)
4–5 years (No, proportion)	60 (3.419%)
5–6 years (No, proportion)	49 (2.792%)
Gender (M, proportion)	936 (53.333%)
BMI, kg/m^2^ (mean, sd)	15.003 ± 2.41
RACHS	
1	73 (4.16%)
2	1268 (72.25%)
3	218 (12.422%)
4	175 (9.915%)
5	21 (1.2%)
PH (No, proportion)	255(14.53%)
Down syndrome (No, proportion)	24(1.368%)
Season of surgery (No, proportion)	
Winter (Dec-Mar)	337 (19.202%)
Spring (Mar-Jun)	505 (28.775%)
Summer (Jun-Sep)	484 (27.578%)
Autumn (Sep-Dec)	429 (24.444%)
Atmospheric pollutants	
AQI (daily average)	116 (81,138)
PM_2.5_ (daily average)	58 (39,89)
PM_10_ (daily average)	118 (84,169)
SO_2_ (daily average)	26 (15,46)
O_3_ (8-h average)	98 (55,150)
T_max_ (daily average)	22 (10,30)
T_min_ (daily average)	10 (-1,19)
Humidity	54 (39,68.75)
VAP (No, proportion)	348 (19.829%)
Mortality (No, proportion)	
Hospital mortality	57 (3.248%)
90 day-mortality	68 (3.87%)
Ventilation (hours)	24 (6, 70)
ICU stay (days)	3.88 (2.0,6.67)
Postoperative hospital stay (days)	9 (7, 13)

Data are median (IQR) or n/N (%)

*VAP* Ventilator-associated pneumonia, *ICU* Intensive care unit, *BMI* Body mass index, *RACHS* Risk Adjustment for Congenital Heart Surgery, *PH* Pulmonary Hypertension, *PM*_*2.5*_ Particulate matter with diameter ≤ 2.5 µm, *PM*_*10*_ Particulate matter with diameter ≤ 10 µm, *SO*_*2*_ Sulfur dioxide, *O*_*3*_ Ozone, *T*_*max*_ Daily maximum temperature of day, *T*_*min*_ Daily minimum temperature of day

**Fig. 2:**
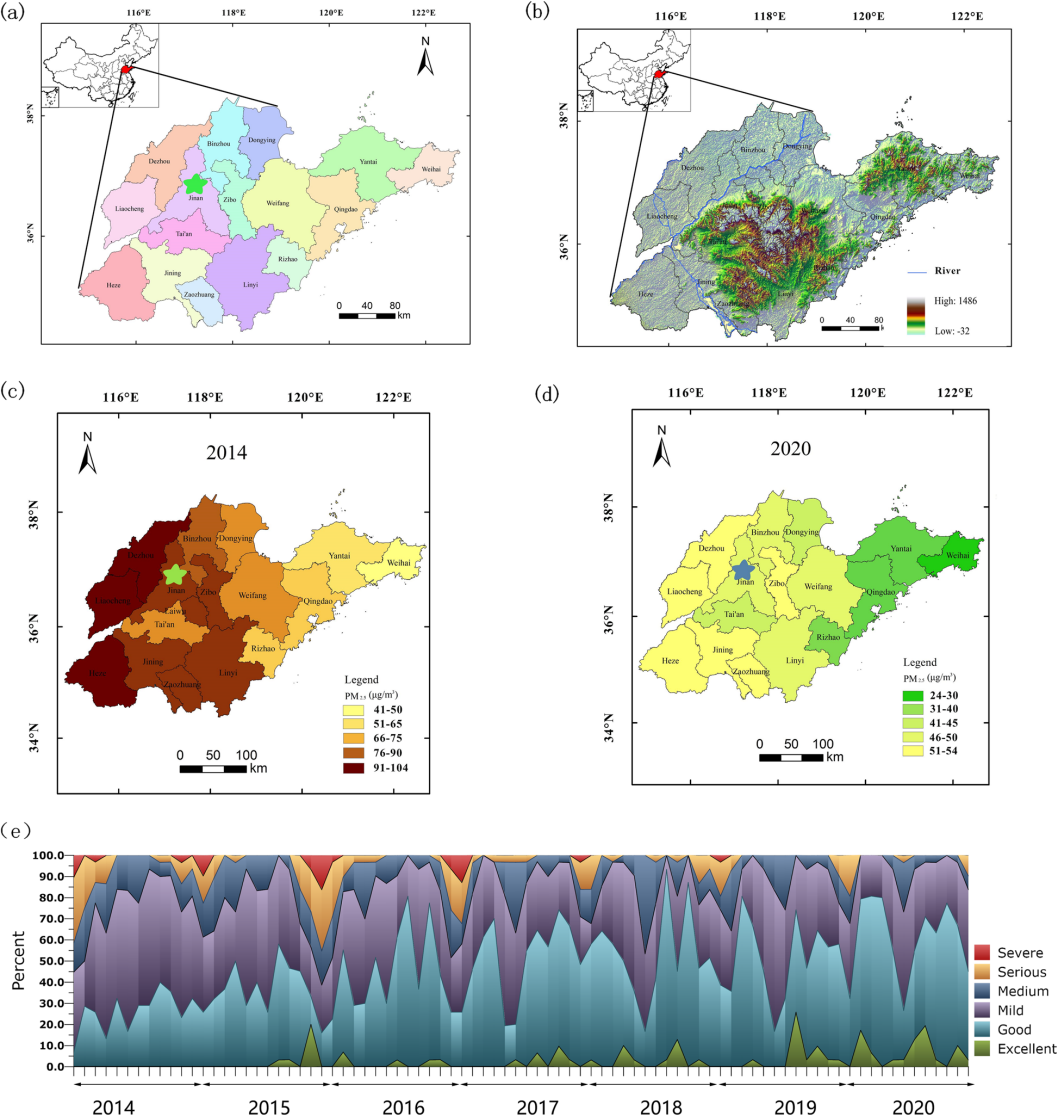
Location of Jinan in China and the air quality for Jinan city from 2014 to 2020. **a** location of Jinan in China; **b** topography of Jinan; **c** annual average PM_2.5_ levels in Jinan in 2014; **d** average PM_2.5_ in Jinan in 2020; **e** air quality in Jinan classified by the AQI from 2014 to 2020. The green star shape represents the location of Jinan in **a**

**Fig. 3:**
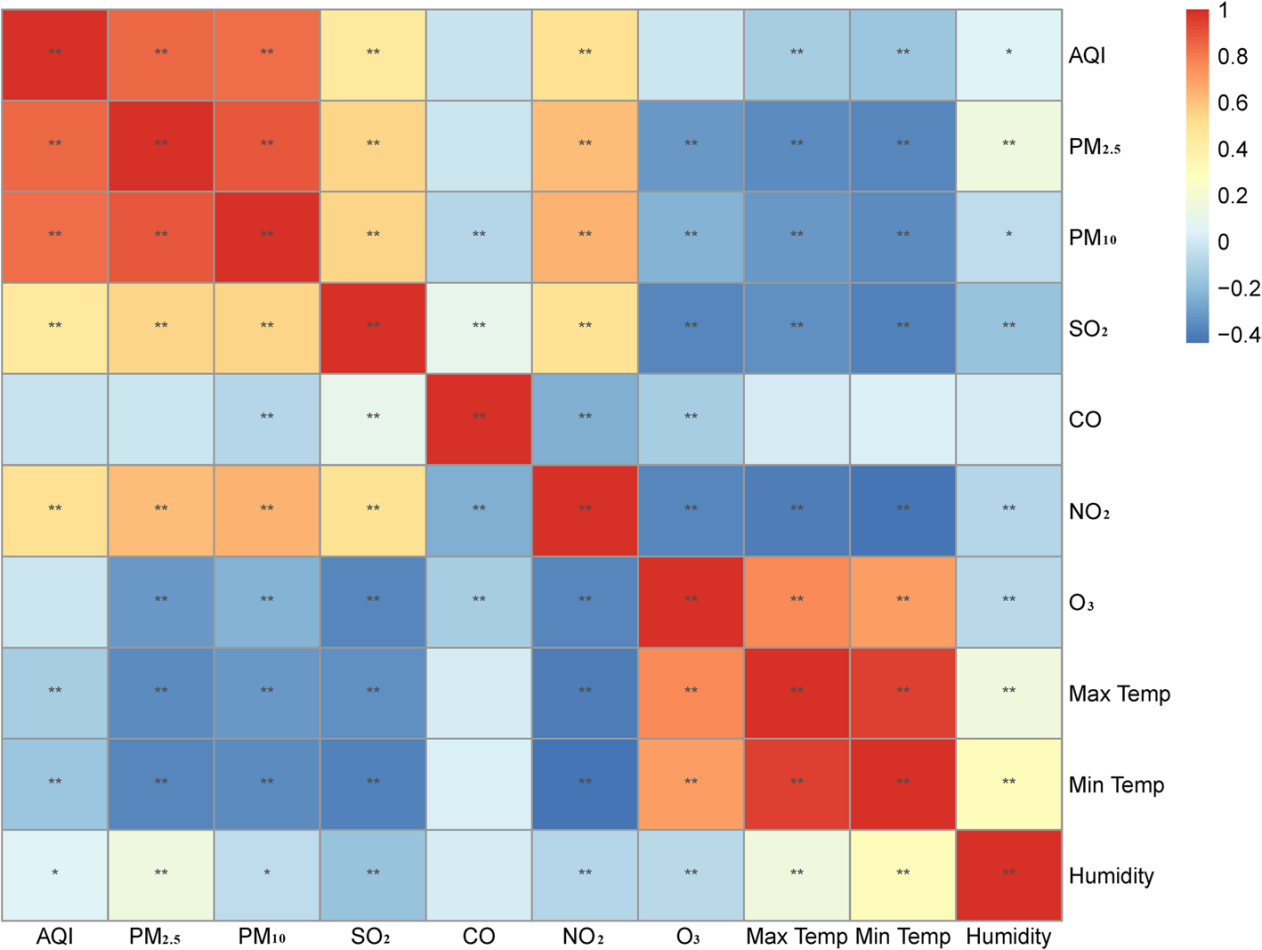
Spearman’s correlation coefficients between daily air pollutants and weather conditions in Jinan, 2014-2020. Note: Max Temp: daily maximum temperature; Min Temp: daily minimum temperature. *˂0.05; **˂0.01

In Jinan, the three main pollutants are PM_2.5_, SO_2_ and O_3_. The air quality for Jinan city from 2014 to 2020 is shown in Fig. 2. During this period, the average concentration of PM_2.5_, PM_10_, O_3_ and SO_2_ were 58, 118, 98 and 26 μg/m^3^ respectively. The daily maximum and minimum temperature and relative humidity were 22 and 10 ˚C and 54%, respectively. However, it was clear that the PM_2.5_ levels in 2014 were significantly worse than those in 2020. During the evaluation period, the daily PM_2.5_ concentration exceeded the current NAAQS of 75 μg/m^3^ on 915 days (35%), and the next NAAQS target of 50 μg/m^3^ on 1581 days (60%). For PM_10_, there were 812 days (31%) on which the levels were more than 150 μg/m^3^. Spearman correlation showed that daily PM_2.5_ was significantly correlated with daily PM_10_ and SO_2_ and O_3_ (Fig. 3 and Suppl. Table S1). We also found a significant correlation between indoor and outdoor PM_2.5_ concentration for the ICU (*r* = 0.8199, *P*˂0.01; Supp. Fig. S2).

**Fig. 4:**
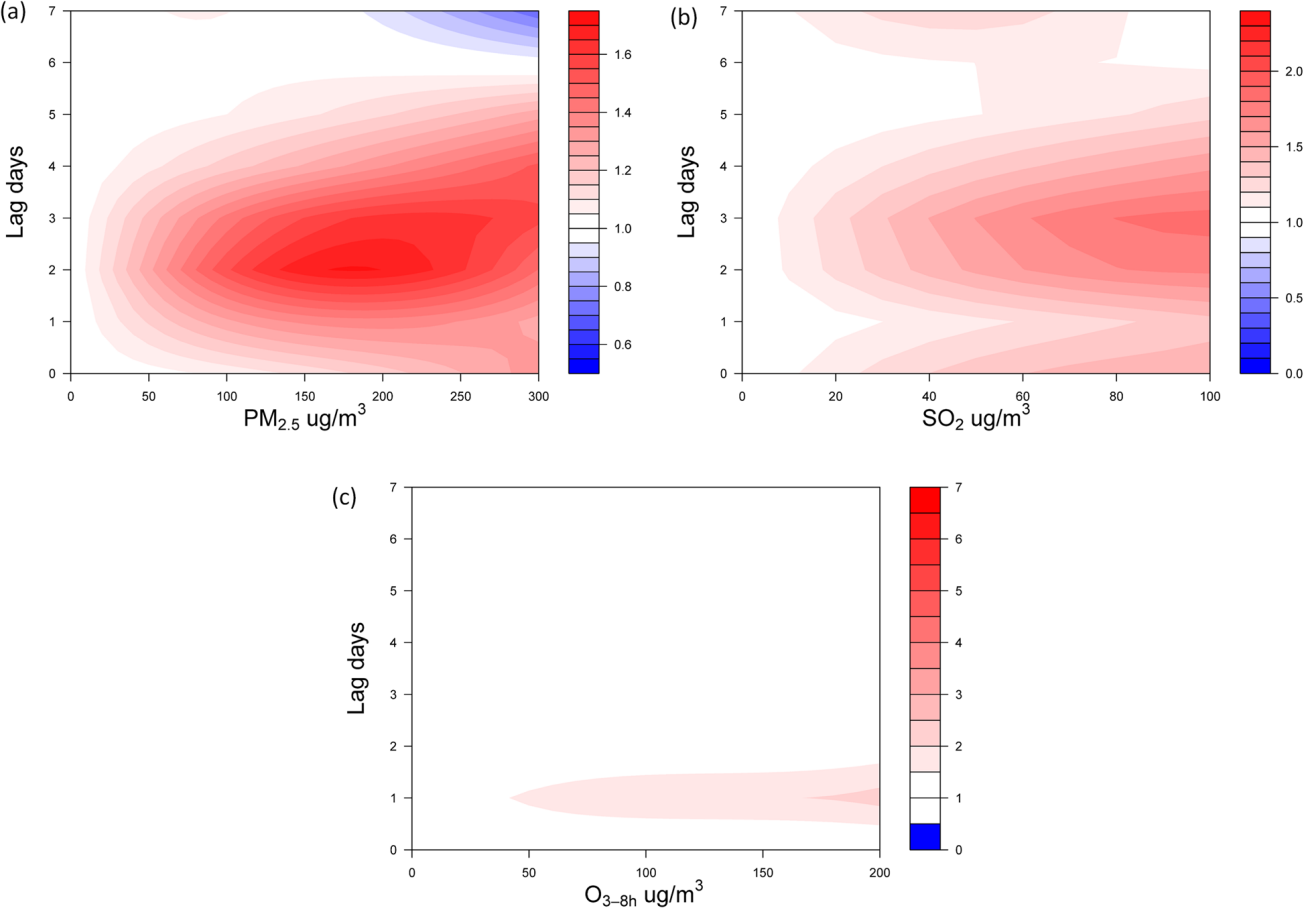
Contour plots showing the adjusted effect of PM_2.5_ (**a**), SO_2_ (**b**) and O_3_ (**c**) on the RR of VAP on different lag days. Notes: RR: relative risk; VAP: ventilator-associated pneumonia

**Fig. 5:**
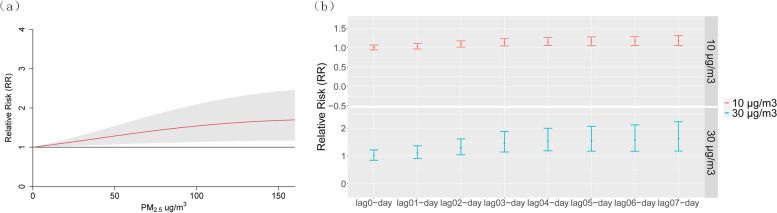
Estimated exposure–response curves for short-term exposures to PM_2.5_. Note: **a** Exposure-repose curve showing the RR increase in VAP with increasing PM_2.5_ concentration. Gray area indicates 95% CIs. **b** Cumulative-repose curve showing the cumulative effect of 10 μg/m^3^ and 30 μg/m^3^ increment of PM_2.5_ on VAP with increasing number of lags days. Lag 0-day is the VAP diagnosis day, lag 01-day indicate the period between VAP diagnosis and 1 day prior, lag 02-day indicate the period between VAP diagnosis and 2 days prior, etc., up to 7 days prior. PM_2.5_: Fine particulate matter; VAP: ventilator-associated pneumonia

To understand how these pollutants may impact the development of VAP, we modeled their effects in DLNMs. All air pollutants affected VAP acutely, with the most significant effects occurring with increased pollutant exposure 2 days prior to VAP diagnosis (lag 2-day) for PM_2.5_ and SO_2,_ while significant risk for O_3_ occurred on lag1-day and only at extremely high levels of this pollutant (Fig. 4). The estimated relative risk (RR) values returned to 1 from lag 4-day. Among the pollutants (PM_2.5_, O_3,_ and SO_2_) evaluated, PM_2.5_ is the most significantly associated with VAP (Fig. 4 and Suppl. Table S2 and Table S3). The maximum effect of PM_2.5_ occurred 2 to 3 days after exposure. A 30 μg/m^3^ increase in PM_2.5_ exposure increased the RR value from 1.016 (95% CI: 0.847–1.22) on lag 0-day to a peak of 1.168 (95% CI: 1.044–1.308) on lag 2-day (Table 2), and the effect was gone by lag 4-day (RR: 1.05; 95% CI: 0.962–1.146). Significant risk for VAP can be seen even with very small increments (10 ug/m^3^) of PM_2.5_ exposure, and there was a dose–response relationship between PM_2.5_ concentration and VAP (Fig. 5A and Fig Supp. S3). While a low 10 μg/m^3^ increment of PM_2.5_ increased the VAP incidence by 5.4% (95% CI: 1.4% ~ 9.5%), a 30 μg/m^3^ increase in PM_2.5_ increased VAP incidence by 16.8% (95% CI:4.4%-30.8%). Interestingly, the lag 0-day to lag 2-day period matches the period of patient admission into the ICU since VAP diagnosis typically occurs 2 days after surgery. Therefore, the indoor ICU air quality may potentially be an underappreciated factor for VAP. As expected, there was also a cumulative effect of PM_2.5_ on VAP. A 30 μg/m^3^ incremental exposure between lag 0-day and lag 7-day (lag 07-day) increased the RR value from 1.016 (95% CI: 0.847–1.22) on lag 0-day to a peak of 1.622 on lag 07-day (95% CI: 1.179–2.232; Fig. 5B).

**Table 2:** Relative risks of developing VAP with combinations of incremental PM2.5 concentrations and lag times

Each PM_2.5_ increase (ug/m^3^)	Lag 0- day	Lag 1-day	Lag 2-day	Lag 3-day	Lag 4-day	Lag 5-day	Lag 6-day	Lag 7-day
10	1.005 (0.945, 1.069)	1.031 (0.971, 1.095)	1.054 (1.014, 1.095)	1.042 (1.006, 1.079)	1.017 (0.987, 1.047)	1.004 (0.971, 1.037)	1.004 (0.979, 1.03)	1.01 (0.96, 1.063)
20	1.011 (0.894, 1.143)	1.062 (0.942, 1.198)	1.11 (1.029, 1.198)	1.085 (1.012, 1.163)	1.033 (0.974, 1.096)	1.008 (0.944, 1.076)	1.008 (0.958, 1.06)	1.02 (0.922, 1.128)
30	1.016 (0.847, 1.22)	1.094 (0.916, 1.307)	1.168 (1.044, 1.308)	1.129 (1.018, 1.251)	1.05 (0.962, 1.146)	1.012 (0.918, 1.115)	1.011 (0.938, 1.09)	1.029 (0.887, 1.195)
40	1.022 (0.804, 1.3)	1.126 (0.891, 1.422)	1.227 (1.058, 1.423)	1.173 (1.025, 1.343)	1.067 (0.952, 1.197)	1.016 (0.894, 1.154)	1.015 (0.919, 1.12)	1.038 (0.853, 1.262)
50	1.028 (0.766, 1.381)	1.157 (0.869, 1.541)	1.286 (1.072, 1.541)	1.217 (1.031, 1.437)	1.085 (0.942, 1.249)	1.021 (0.873, 1.193)	1.018 (0.902, 1.148)	1.045 (0.823, 1.327)
60	1.035 (0.733, 1.462)	1.187 (0.848, 1.66)	1.343 (1.086, 1.661)	1.261 (1.038, 1.531)	1.102 (0.935, 1.299)	1.026 (0.854, 1.232)	1.021 (0.887, 1.175)	1.05 (0.795, 1.388)

Distributed lag non-linear model was fitted by incorporating the daily average PM_2.5_ on the lag days, covariates including maximum and minimum temperature, and humidity. PM_2.5_ value and its lag effect were modelled by a polynomial function with 3 degrees of freedom. Data presented as risk score (95% confidence interval)

To confirm the effect of PM_2.5_ alone and in combination with other pollutants, we fixed the lag time at 2 days prior to VAP (lag 2-day), incremental PM_2.5_ exposure at 10 ug/m^3^, and modeled the effect of PM_2.5_ alone (single pollutant analysis), with SO_2_ (PM_2.5_ + SO_2_), with O_3_ (PM_2.5_ + O_3_), and with both pollutants (main analysis; Table 3). All pollutant combinations significantly increased the risk for VAP, with PM_2.5_ alone showing the lowest RR value at 1.054 (95%CI 1.014–1.095). Addition of SO_2_ or O_3_ increased the RR value to 1.055 (95% CI 1.015–1.096 for SO_2_; 1.016–1.096 for O_3_), while addition of both pollutants increased the RR value further to 1.056 (95%CI:1.016–1.097). Thus, the pollutants show incremental risk for VAP when presented together. Surprisingly, modeling of a low-exposure model (PM_2.5_ ˂50 ug/m^3^ at lag2-day) showed a higher RR score of 1.117 (95% CI: 1.045–1.195).

**Table 3:** Relative risk of developing VAP with exposure to different combination of pollutants

	Increase in VAP (%)	SE	P	95% CI	RR
Main analysis^a^	5.4	0.019	0.005	1.016–1.097	1.056
Single-pollutant analysis^b^	5.3	0.02	0.007	1.014–1.095	1.054
Two-pollutant model^c^					
PM_2.5_ + SO_2_	5.3	0.02	0.006	1.015–1.096	1.055
PM_2.5_ + O_3_	5.4	0.019	0.006	1.016–1.096	1.055
Low-exposure analysis^d^	11.1	0.034	0.001	1.045–1.195	1.117
High-exposure analysis^d^	2.4	0.023	0.295	0.979–1.072	1.025

For this analysis, lag time was fixed at lag 2-day and the incremental PM_2.5_ level at 10 μg/m^3^

^a^The main analysis modeled the effects of all three pollutants, PM_2.5_, SO_2_ and O_3_

^b^The single-pollutant analysis modeled the effect of PM _2.5_ alone

^c^The two-pollutant analysis modeled the effect of PM _2.5_ with SO_2_ or O3

^d^According to the recent Chinese guideline for PM_2.5_, the cut-off value for low- and high-exposure is 50 μg/m^3^

**Fig. 6:**
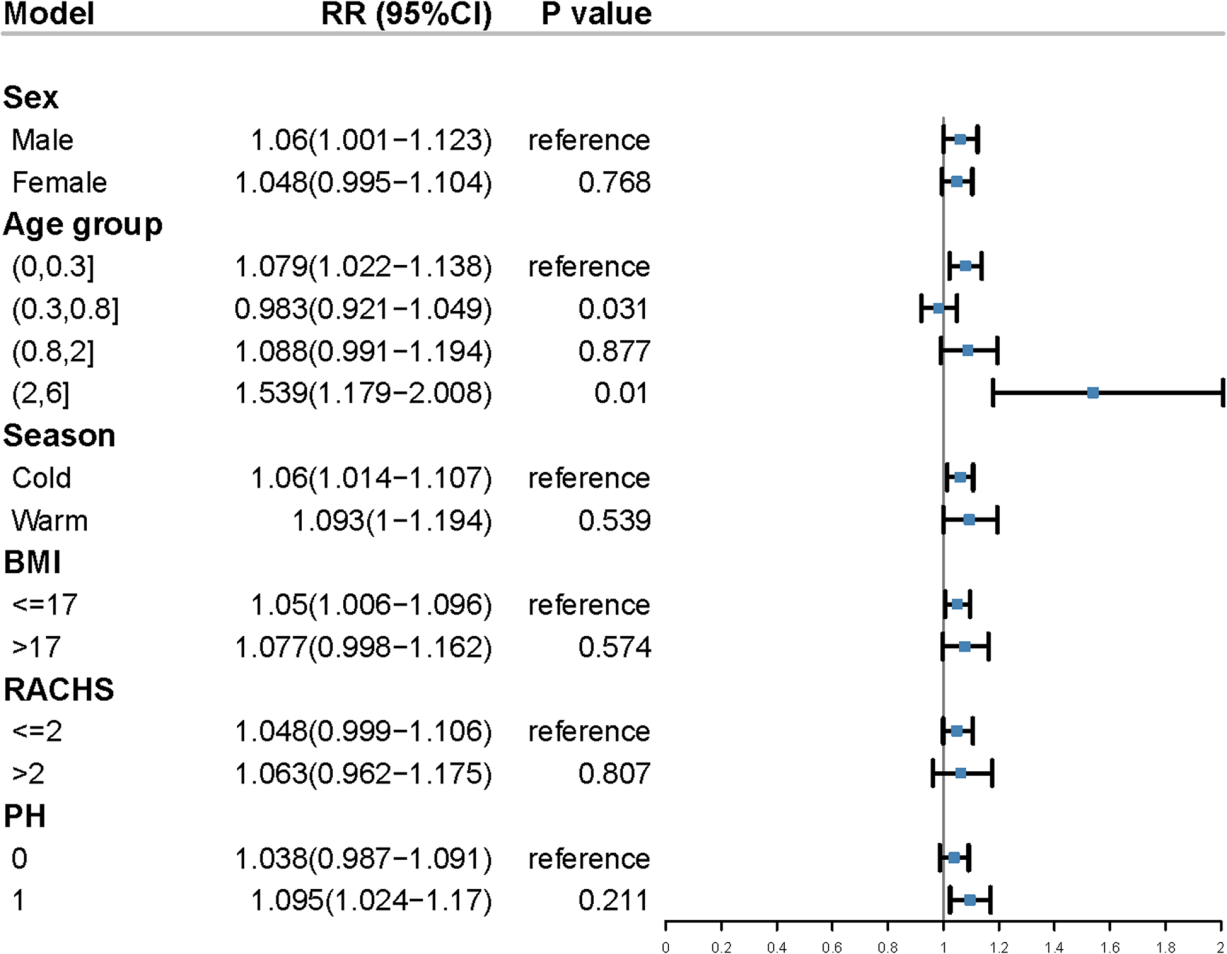
Modeling the effect of 10 μg/m^3^ increase in PM_2.5_ on the relative risk of developing VAP in subgroups of child patients. Notes: The reference groups were used when assessing effect modifications. Statistical significance is defined as *P* < 0.05 compared with the reference group. BMI: body mass index; RACHS: Risk Adjustment for Congenital Heart Surgery; PH: pulmonary hypertension

To determine whether other clinical parameters can affect the relative risk of PM_2.5_ on VAP, we analyzed parameters including patient gender, age and BMI score, season in which surgery took place, patients’ RACHS classification and presence of pulmonary hypertension (PH). We found that age, particularly the 0–0.3 years age group, was significantly more susceptible to VAP development with increased PM_2.5_ exposure (RR 1.079; 95% CI: 1.022–1.138). A slight reduction in RR was found in the 0.3–0.8 years age group (RR 0.983; 95% CI: 0.921–1.049). The 2–6 years old group also showed statistically significant association (Fig. 6). However, retrospective analysis of this patient group showed that they were mainly re-operated patients with complex cardiac malformation and long tracheal intubation times. Therefore, results for the 2–6 years old patients cannot be interpreted in the context of PM_2.5_ and VAP. Patients with a low BMI value (˂17) also showed enhanced risk for VAP with increased PM_2.5_ exposure (RR:1.05; 95% CI: 1.006–1.096). As expected, PH also significantly increases the effect of PM_2.5_ on VAP, indicating that PM_2.5_ can further aggravate VAP in patients with severe cardiac anomalies.

## Discussion

Relationships short-term pollutant exposure and mortality in hospitalized patients has been demonstrated previously, but effects on specific populations of patients are less well understood. With increasing appreciation of high-risk groups such as children, the current report is the first large scale study on the acute effects of PM_2.5_ on VAP in child patients. We observed for the first time a delayed and cumulative effect of PM_2.5_ exposure on ICU outcome. An increase of as little as 10 μg/m^3^ of PM_2.5_ was sufficient to increase the relative risk for VAP. The effect was delayed, with the RR plateauing for exposures 2 to 3 days prior to VAP diagnosis. Earlier exposure, 4 days or more before VAP diagnosis, presented only baseline risk. Our results also provide evidence that even at levels lower than the current NAAQS, PM_2.5_ remains a significant risk for VAP. These results suggest that short-term exposure to even low levels of PM_2.5_ increases the risk of VAP in vulnerable individuals.

Currently, there are no research on the effect of ambient air pollution in heavily polluted regions on critical ICU patients such as those who have undergone major surgeries. Thus, our study is timely and will contribute significantly to the management of public health. Many studies on the effects of air pollutants on respiratory diseases are conducted in specific locations, and are therefore, subjected to the particular climate in that region and the susceptibility of the local population. Regional climates can influence the local air pollutant concentrations and composition. Jinan is an industrial city heavily focused on manufacturing and combined with the standard practice of burning coal in the winter put this city among the top 10 most polluted cities in China. PM_2.5_ is the primary pollutant in Jinan. In 2013, the annual average PM_2.5_ concentration exceeded twice the WHO recommended levels, with the National Standard 24-h mean level exceeded on 235 days of the year [24]. Our study showed that the average PM_2.5_ level was 90 μg/m^3^ in 2014 and gradually declined to 58 μg/m^3^ by 2020. But this is still higher than the WHO interim target 3 level of 37.5 μg/m^3^. Recent studies conducted in Jinan found significant increase in pediatric pneumonia hospitalization rate with elevated PM_2.5_ concentrations [24]. While other heavily polluted cities such as Shanghai, Guangzhou and Shenzhen, also demonstrated increased risk for respiratory diseases [25, 26]. Thus, the findings in our study are not only relevant for Jinan but may serve as benchmarks for other heavily polluted cities.

The ICU houses the most vulnerable patients in the most critical conditions; thus, it is often perceived as a protective shield against stressors and harmful environmental influences. However, the ability of ambient pollution to compromise the function of the ICU is underappreciated. Pre-admission air pollution exposure can aggravate the critical conditions of patients in the ICU. Groves et al. reported that pre-admission exposure to PM_2.5_ is positively correlated with ICU mortality within 30 days of admission [27]. While Weerdt et al. found that ICU patients with increased exposure to air pollution before admission were more likely to require longer duration of ventilation [28]. The ICU ventilation system normally filters the incoming air and combined with the spatial separation of this unit supports a sterile image of the ICU. However, this may be far from the truth. Wolf et al. reported significant variation in the indoor air quality of an ICU and more PM_2.5_ and volatile organic compounds accumulate in neonatal incubators compared to the surrounding air [29]. Similarly, after monitoring our ICU for a year, both inside and outside, we found that on sunny days, the PM_2.5_ concentrations were similar in both locations, while indoor PM_2.5_ levels were actually higher on cloudy and rainy days. This evidence strongly suggests that ICUs should be compulsorily equipped with more advanced air filtration systems such as laminar airflow ventilation systems (LAF), to achieve a lower PM_2.5_ concentration and reduce respiratory infections in the ICU.

While LAF systems seem attractive in many aspects, more research is required in this area. Particulate matters are critical determinants of airborne bacterial concentration and community structure. While LAF systems are 89% more effective than displacement ventilation systems in reducing air contamination [30], use of LAF in operation rooms were associated with significantly increased infection following total knee arthroplasty in New Zealand over a 10-year period [31]. However, we found that conventional ventilation systems could not reduce indoor PM_2.5_ concentration in the ICU, and patients are not receiving optimal quality air with very a low level of microbial colony forming units. Improving air qualities will gain more traction in the future as increasing climate changes and aging populations will put more people, both young and old, at risk of air pollution-related respiratory diseases. However, there are no clear standards for maintaining ICU ventilation systems and the responsibility linked to their function and maintenance is similarly vague. With continuous technological advancements, clear and consistent universal standards are required to guide the appropriate use of such high-quality air-conditioning systems to control VAP in the ICU [32].

Our results showed that the effects of air pollution on VAP incidence is acute, delayed by 2–3 days and cumulative. This is consistent with previous studies where a range of lag days have been demonstrated. In association with emergency department visits for asthma, PM_2.5_ showed no delay (lag 0-day) [25]; a 3–4 day lag for the maximum effects of PM_2.5_ on the risk of pneumonia [20]; a 2-day delay for ambient air pollution on hospitalization for respiratory diseases [33]. Similarly, the cumulative effect over 7 days that we observed in this study is also consistent with the cumulative effects of O_3_ on lower respiratory diseases in children over 6 days [34], and the cumulative-day lag effect of PM_2.5_ on asthma [25]. In our data, PM_2.5_ posed the most significant risk for VAP when pre-exposure occurred 2 days prior to VAP diagnosis. Several mechanisms may contribute to this lag pattern: first, we speculate that the mechanism by which PM_2.5_ can aggravate VAP is not by the carrying mode of bacteria, but due to interference caused by PM_2.5_ to the functions of the human lung and multiple organ systems. The bacteria (*Enterobacteriaceae* and *Staphylococcus aureus*) have similar sizes to PM_2.5_, almost at the µm level. The smaller the size of the inhaled particle, the deeper it can penetrate into human respiratory system, leading to inflammatory reactions at the alveolar level and resulting in cytotoxicity and dysfunction [35]. Thus, ambient air pollution constitutes a serious risk factor not only for respiratory infections, but also for the development of reduced pulmonary function and/or aggravation of existing pulmonary disease [36]. Secondly, multiple system and organ disorders as well as inflammation arising from the cardiopulmonary bypass of surgery also takes time to develop [37]. Patients typically develop dyspnea, fever, cough and phlegm one or two days after surgery. Fine particulate matters can trigger production of inflammatory molecules over time. PM_10_ and ultrafine particles have been shown to stimulate C-reactive protein and ICAM-1 in the blood after 1–2 days exposure [38], and systemic inflammatory biomarkers were detected after exposure to increased levels of ambient air pollution for a few days [39]. Thirdly, pediatric population often have immature respiratory systems, higher breathing rate, and underdeveloped immune function, increasing their susceptibility to respiratory infections. Air pollutants are thus more likely to steadily aggravate their sensitive lungs during the 2 days post-surgery and trigger VAP.

The current NAAQS in China for daily PM_2.5_ is 75 μg/m^3^. Interestingly, we found that PM_2.5_ levels below 50 μg/m^3^ increased the risk for VAP as higher levels of PM_2.5_. One study showed that even PM_2.5_ levels below 15 μg/m^3^ can increase the mortality rate of a population [40]. These results indicate that even at levels well below the current standards, PM_2.5_ is still an important health risk. Previous studies generally showed a linear relationship between PM_2.5_ concentration and mortality [41]. The exposure–response curve in our study also showed a similar linear relationship with a shallow slope between PM_2.5_ and VAP.

We found that gender can significantly impact the association between PM_2.5_ and VAP where boys are slightly more at risk to develop VAP after PM_2.5_ exposure than girls. Similar gender bias was observed in association studies between ambient air pollution and outpatient visits for respiratory diseases [42]. Age is another factor that influences the effect of PM_2.5_ on VAP development with younger patients at higher risks. Patients with pulmonary hypertension (PH) were also more likely to develop VAP with increased PM_2.5_ exposure. Young patients receiving surgery are generally presented with complex cardiac malformations, immature lungs, accompanied with low BMI and/or serious complication such as PH, making this group particularly vulnerable to VAP. Children age 0–5 years were shown to be more susceptible to acute upper respiratory tract infections than those age 6–14 years with short-term exposure to air pollution, while low BMI increases the risk for surgical site infection and antibiotic usage [43]. Our study also supports that young age, low BMI and accompanied PH increase the susceptibility of a population to VAP after exposure to PM_2.5_.

Our study has four strengths. First, we simulated the interaction between PM_2.5_ and VAP with DLNM which provides a more accurate estimation of the interactions since a number of factors including time-course, exposure–response relationship, and multifactor interaction can be taken into account while controlling for exposures during different time periods simultaneously [44, 45]. The DLNM also help to account for the phenomenon of “harvest” where VAP may occur in vulnerable subjects immediately or delayed after exposure, thus reducing the number of subjects at risk and the overall long-term impact of PM_2.5_ [46, 47]. Second, this is the largest study to date on the impact of PM_2.5_ on VAP development in child patients. Since all patients were operated on by the same experienced group of surgeons, interference from surgical techniques is significantly reduced. Third, we evaluated air pollution data over a long period spanning more than 7 years and monitored the air quality in and around the ICU for a whole year. These data are therefore likely to be reliable in determining the association between PM_2.5_ and VAP. Fourth, the effect of air pollution on VAP was studied in a heavily polluted city of eastern China, in an ICU equipped with conventional ventilation systems. Our data may be relevant for ICU construction in developing countries.

However, several limitations are associated with this study. First, this study focused on the acute effect of air pollution on pediatric patients undergoing cardiac surgery in a single center, which limits the generalizability of the findings to other child populations and does not address the long-term effect of air pollution on VAP risk. We will try to implement a prospective multicenter or multi-PICU cohort study and provide higher level of evidence in the future clinical practice. Furthermore, the effects of long-term exposure to fine particulate matter on VAP are still unclear and thus the investigation of the long-term influence of PM_2.5_ exposure on VAP is meaningful. Second, we have used the average air qualities from 11 monitoring sites to represent the ambient pollutant level. Due to regional variations, the true exposure level for each patient is likely to vary depending on their actual location. Thus, some measurement errors for individual exposure levels are to be expected. Besides, the most recent data used in this study were from almost 2 years ago; more current data, especially from the covid-19 period when significant decrease in air pollution was observed in many regions, may provide more insights into the effect of PM_2.5_ on VAP development. Third, the patients’ diagnoses were obtained from the ICD10 coding at the time of discharge, which may potentially contain misclassification of the type of respiratory disease, particularly when diagnosed by different clinicians. Furthermore, we could not collect the complete hospitalization information of patients, such as the detailed ventilator settings, due to inadequate electronic medical records. Fourth, many meteorological factors could impact the PM concentrations, including the dispersion, growth, chemical production, photolysis, and deposition of PM. In our DLNM model, we have controlled the most important confounding values (daily maximum and minimum temperatures and humidity) to test the stability of the results. In the future study, we will investigate the effects of multiple meteorological factors, such as cloud cover, temperature, wind, pressure, precipitation, rainfall, radiation, and the planetary boundary layer height.

## Conclusion

In line with the growing body of evidence, we show here that short-term PM_2.5_ exposure increases the risk of VAP development among children hospitalized in the ICU. This risk showed delayed and cumulative effects, suggesting that an improvement in the air quality before and during ICU stay can influence respiratory infection. PM_2.5_ poses risk even when present at levels well below the current national air quality standards, indicating that these standards could be revised for susceptible populations.

## Supplementary Information

**Additional file 1: Table S1.** Spearman correlation coefficients of daily number concentrations of particles with daily mass concentrations of air pollutants and weather conditions. **Table S2.** Relative risks of VAP at specific combinations of O_3_ values and lag times. **Table S3.** Relative risks of VAP at specific combinations of SO_2_ values and lag times. **Figure S1.** The criteria for diagnosing Ventilator-Associated Pneumonia. **Figure S2.** The indoor and outdoor PM_2.5_ concentration in ICU during 2020. **Figure S3.** Estimated exposure-response curves for short-term exposures to PM_2.5_.

## Data Availability

The datasets used and/or analyzed during the current study are available from the corresponding author on reasonable request.

## Abbreviations

ICU: Intensive Care Unit
VAP: Ventilator-associated pneumonia
PM_2.5_: Fine particulate matter
SO_2_: Sulfur dioxide
O_3_: Ozone
NO_2_: Nitrogen dioxide
WHO: World Health Organization
IT-1: Interim Target 1
NAAQS: National Ambient Air Quality Standards
BMI: Body mass index
DLNM: Distributed lag non-linear model
GDP: Gross domestic product
AQI: Air quality index
SD: Standard deviation
SE: Standard error
IQR: Inter-quartile-range
RR: Relative risk
df: Degrees of freedom
DOW: Day of the week
(T_MAX_) and (T_MIN_): Maximum and minimum temperatures of the day
RACHS: Risk adjustment for congenital heart surgery
PH: Pulmonary hypertension
