# Supplementary material for: Short-term exposure to ambient fine particulate pollution aggravates ventilator-associated pneumonia in pediatric intensive care patients undergoing cardiovascular surgeries
Source: Environ Health. 2023 Apr 26;22:39. doi: 10.1186/s12940-023-00991-y (PMC10132412; doi:10.1186/s12940-023-00991-y)
Supplement: Supplementary file 1 — Additional file 1: Table S1. Spearman correlation coefficients of daily number concentrations of particles with daily mass concentrations of air pollutants and weather conditions. Table S2. Relative risks of VAP at specific combinations of O3 values and lag times. Table S3. Relative risks of VAP at specific combinations of SO2 values and lag times. Figure S1. The criteria for diagnosing Ventilator-Associated Pneumonia. Figure S2. The indoor and outdoor PM2.5 concentration in ICU during 2020. Figure S3. Estimated exposure-response curves for short-term exposures to PM2.5. [file 12940_2023_991_MOESM1_ESM.docx]

**Supplementary Appendix**

This appendix has been provided by the authors to give readers additional information about their work.

**Short-term exposure to ambient fine particulate pollution** **aggravates ventilator-associated pneumonia** **in pediatric intensive care patients undergoing cardiovascular surgeries**

Zhaomei Cui ^1^^#^, Yingying Ma^2#^, Yuanyuan Yu^3#^, Na Li^4^, Jun Wang^5^, Anbiao Wang ^1^, Qi Tan ^1*^

Contents

[Table S1: Spearman correlation coefficients of daily number concentrations of particles with daily mass concentrations of air pollutants and weather conditions 2](#_Toc129813987)

[Table S2. Relative risks of VAP at specific combinations of O_3_ values and lag times. 3](#_Toc129813988)

[Table S3. Relative risks of VAP at specific combinations of SO_2_ values and lag times. 4](#_Toc129813989)

[Determine the variables which have the potential to affect VAP 5](#_Toc129813990)

[Figure S1: The criteria for diagnosing Ventilator-Associated Pneumonia 6](#_Toc129813991)

[Figure S2: The indoor and outdoor PM_2.5_ concentration in ICU during 2020 7](#_Toc129813992)

[Figure S3: Estimated exposure-response curves for short-term exposures to PM_2.5_ 8](#_Toc129813993)

[References: 9](#_Toc129813994)

Table S1: Spearman correlation coefficients of daily number concentrations of particles with daily mass concentrations of air pollutants and weather conditions.

A: shown r value

|  | AQI | PM_2.5_ | PM_10_ | SO_2_ | CO | NO_2_ | O_3_ | Tmax | Tmin | humidity |
| --- | --- | --- | --- | --- | --- | --- | --- | --- | --- | --- |
| AQI | 1 | 0.848 | 0.839 | 0.448 | -0.031 | 0.506 | -0.006 | -0.123 | -0.149 | 0.044 |
| PM_2.5_ |  | 1 | 0.888 | 0.551 | -0.008 | 0.615 | -0.315 | -0.362 | -0.367 | 0.146 |
| PM_10_ |  |  | 1 | 0.549 | -0.084 | 0.646 | -0.226 | -0.308 | -0.359 | -0.056 |
| SO_2_ |  |  |  | 1 | 0.107 | 0.486 | -0.378 | -0.349 | -0.381 | -0.174 |
| CO |  |  |  |  | 1 | -0.243 | -0.127 | 0.011 | 0.03 | 0.022 |
| NO_2_ |  |  |  |  |  | 1 | -0.368 | -0.401 | -0.436 | -0.09 |
| O_3_ |  |  |  |  |  |  | 1 | 0.761 | 0.701 | -0.07 |
| Tmax |  |  |  |  |  |  |  | 1 | 0.956 | 0.158 |
| Tmin |  |  |  |  |  |  |  |  | 1 | 0.302 |
| humidity |  |  |  |  |  |  |  |  |  | 1 |

B: shown *p* value

|  | AQI | PM_2.5_ | PM_10_ | SO_2_ | CO | NO_2_ | O_3_ | Tmax | Tmin | humidity |
| --- | --- | --- | --- | --- | --- | --- | --- | --- | --- | --- |
| AQI | <0.001 | <0.001 | <0.001 | <0.001 | 0.118 | <0.001 | 0.755 | <0.001 | <0.001 | 0.027 |
| PM_2.5_ |  | <0.001 | <0.001 | <0.001 | 0.707 | <0.001 | <0.001 | <0.001 | <0.001 | <0.001 |
| PM_10_ |  |  | <0.001 | <0.001 | <0.001 | <0.001 | <0.001 | <0.001 | <0.001 | 0.005 |
| SO_2_ |  |  |  | <0.001 | <0.001 | <0.001 | <0.001 | <0.001 | <0.001 | <0.001 |
| CO |  |  |  |  | <0.001 | <0.001 | <0.001 | 0.569 | 0.137 | 0.266 |
| NO_2_ |  |  |  |  |  | <0.001 | <0.001 | <0.001 | <0.001 | <0.001 |
| O3 |  |  |  |  |  |  | <0.001 | <0.001 | <0.001 | <0.001 |
| Tmax |  |  |  |  |  |  |  | <0.001 | <0.001 | <0.001 |
| Tmin |  |  |  |  |  |  |  |  | <0.001 | <0.001 |
| humidity |  |  |  |  |  |  |  |  |  | <0.001 |

Notes: PM_2.5_, particulate matter with an aerodynamic≤2.5 μm; SO_2_, sulfur dioxide; NO_2_, nitrogen dioxide; CO, carbon monoxide; O3, ozone; Tmax, daily maximum temperature; Tmin, daily minimum temperature

,

## Table S2. Relative risks of VAP at specific combinations of O_3_ values and lag times.

|  | Lag 0-day | Lag 1-day | Lag 2-day | Lag 3-day | Lag 4-day | Lag 5-day | Lag 6-day | Lag 7-day |
| --- | --- | --- | --- | --- | --- | --- | --- | --- |
| 10 | 1 (0.783, 1.277) | 1.113 (0.88, 1.408) | 0.975 (0.847, 1.121) | 0.909 (0.773, 1.069) | 0.94 (0.833, 1.06) | 0.991 (0.843, 1.164) | 0.995 (0.887, 1.116) | 0.966 (0.779, 1.197) |
| 20 | 0.996 (0.625, 1.589) | 1.235 (0.788, 1.936) | 0.952 (0.728, 1.244) | 0.831 (0.609, 1.134) | 0.89 (0.707, 1.12) | 0.987 (0.725, 1.343) | 0.994 (0.799, 1.237) | 0.934 (0.619, 1.408) |
| 30 | 0.985 (0.516, 1.882) | 1.36 (0.729, 2.537) | 0.933 (0.644, 1.352) | 0.769 (0.5, 1.182) | 0.854 (0.621, 1.174) | 0.995 (0.649, 1.525) | 1.001 (0.74, 1.353) | 0.906 (0.513, 1.6) |
| 40 | 0.963 (0.447, 2.076) | 1.482 (0.708, 3.104) | 0.919 (0.593, 1.425) | 0.724 (0.435, 1.205) | 0.836 (0.574, 1.217) | 1.02 (0.615, 1.692) | 1.019 (0.715, 1.454) | 0.884 (0.451, 1.732) |
| 50 | 0.933 (0.405, 2.147) | 1.596 (0.716, 3.556) | 0.911 (0.567, 1.464) | 0.693 (0.399, 1.205) | 0.834 (0.556, 1.252) | 1.062 (0.614, 1.837) | 1.049 (0.717, 1.536) | 0.866 (0.418, 1.794) |
| 60 | 0.898 (0.379, 2.129) | 1.698 (0.744, 3.878) | 0.908 (0.558, 1.477) | 0.674 (0.381, 1.191) | 0.844 (0.556, 1.281) | 1.116 (0.635, 1.962) | 1.086 (0.736, 1.602) | 0.854 (0.404, 1.804) |

Notes: VAP: ventilator-associated pneumonia, O_3_: ozone

### Table S3. Relative risks of VAP at specific combinations of SO_2_ values and lag times.

|  | lag0-day | lag 1-day | lag2-day | lag3-day | lag4-day | lag5-day | lag6-day | lag7-day |
| --- | --- | --- | --- | --- | --- | --- | --- | --- |
| 10 | 1.084 (0.905, 1.298) | 1.032 (0.883, 1.206) | 1.116 (1.013, 1.23) | 1.131 (1.015, 1.26) | 1.067 (0.985, 1.156) | 1.014 (0.91, 1.13) | 1.03 (0.948, 1.119) | 1.093 (0.929, 1.286) |
| 20 | 1.164 (0.828, 1.636) | 1.065 (0.794, 1.429) | 1.232 (1.026, 1.479) | 1.263 (1.029, 1.549) | 1.134 (0.975, 1.319) | 1.03 (0.839, 1.265) | 1.056 (0.903, 1.234) | 1.172 (0.864, 1.59) |
| 30 | 1.237 (0.769, 1.991) | 1.099 (0.729, 1.658) | 1.341 (1.039, 1.73) | 1.388 (1.043, 1.847) | 1.199 (0.971, 1.481) | 1.049 (0.788, 1.398) | 1.076 (0.865, 1.338) | 1.227 (0.804, 1.874) |
| 40 | 1.301 (0.723, 2.342) | 1.134 (0.682, 1.885) | 1.439 (1.05, 1.973) | 1.503 (1.055, 2.14) | 1.262 (0.972, 1.64) | 1.071 (0.752, 1.527) | 1.091 (0.834, 1.427) | 1.256 (0.747, 2.11) |
| 50 | 1.355 (0.688, 2.67) | 1.17 (0.65, 2.104) | 1.526 (1.06, 2.195) | 1.604 (1.065, 2.414) | 1.323 (0.978, 1.79) | 1.096 (0.728, 1.65) | 1.1 (0.808, 1.499) | 1.257 (0.694, 2.278) |
| 60 | 1.398 (0.661, 2.959) | 1.206 (0.63, 2.309) | 1.598 (1.069, 2.389) | 1.689 (1.074, 2.656) | 1.381 (0.988, 1.93) | 1.124 (0.715, 1.766) | 1.104 (0.786, 1.553) | 1.233 (0.643, 2.366) |

Notes: VAP: ventilator-associated pneumonia, SO_2_: sulfur dioxide

#### Determine the variables which have the potential to affect VAP

We enrolled 20 variables for the risk factors for VAP according the literature[1-5] and our experiences. They are: sex, age, BMI, the season during which the surgery was conducted (warm and cold seasons), risk adjustment for congenital heart surgery (RACHS) classification, whether pulmonary hypertension; Use of cardiopulmonary bypass, Duration of cardiopulmonary bypass, Aortic cross-clamp time; Prior antibiotics, Previous operation, ambient air pollution exposure assessment (PM_2.5_, PM_10_, SO_2_, O_3_, NO_2_) and meteorological conditions (maximum and minimum temperature, humidity).

#### Figure S1: The criteria for diagnosing Ventilator-Associated Pneumonia


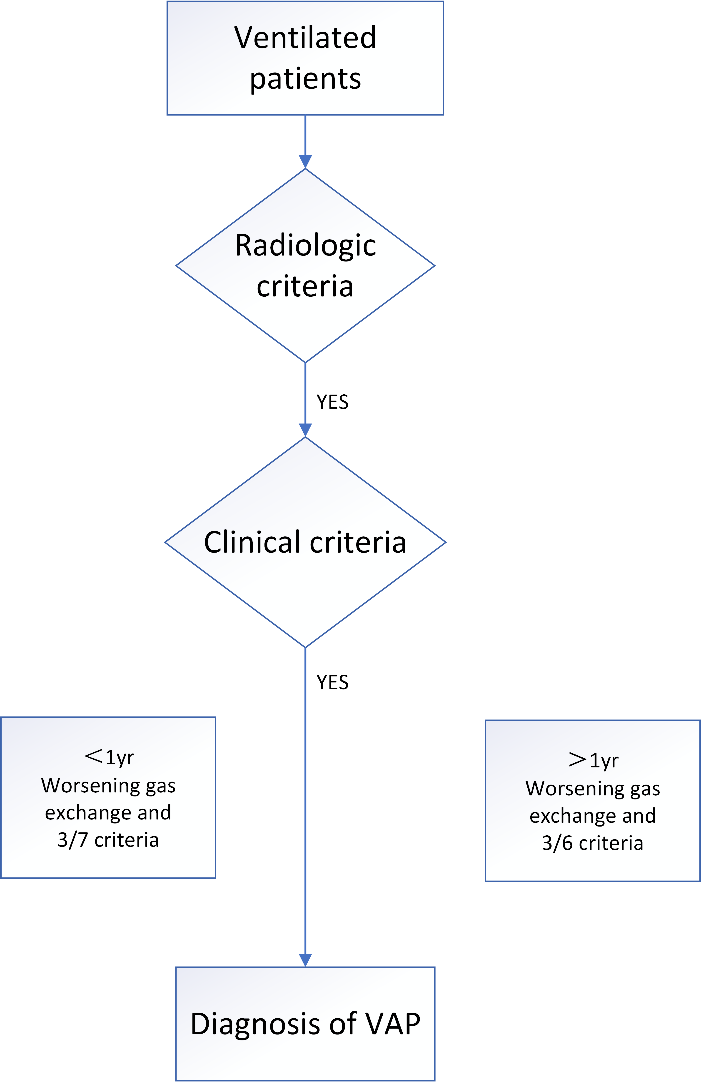


Note: In our hospital, the diagnosis of VAP is reference from Centers for Disease Control and Prevention[6]: Radiologic Criteria: ≥2 imaging test results with one of the following: New & Persistent or Progressive & persistent: infiltrate or consolidation or cavitation or pneumatoceles. Clinical Criteria: Age < 1 year: 1. Temperature instability, with ≥37.5℃ or <36.0℃; 2. Leukopenia (≤4000 WBC/mm^3^) or leukocytosis (≥15,000 WBC/mm^3^) and left shift (≥10% band forms); 3. New onset or change in character of purulent sputum or increased respiratory secretions or increased suctioning requirements; 4. Apnea, tachypnea, nasal flaring with retraction of chest wall or grunting; 5. Wheezing, rales or rhonchi; 6. Cough; 7. Bradycardia (<100 bts/min) or tachycardia (>170 bts/min). Age > 1 years or ≤12 years: 1. Fever (>38.0℃) or hypothermia (<36.0℃); 2. Leukopenia (≤4000 WBC/mm^3^) or leukocytosis (≥15,000 WBC/mm^3^); 3. New onset or change in character of purulent sputum or increased respiratory secretions or increased suctioning requirements; 4. New onset or worsening cough or dyspnea or tachypnea; 5. Rales or bronchial breath sounds; 6. Worsening gas exchange. VAP: ventilator-associated pneumonia.

###### Figure S2: The indoor and outdoor PM_2.5_ concentration in ICU during 2020

(a)


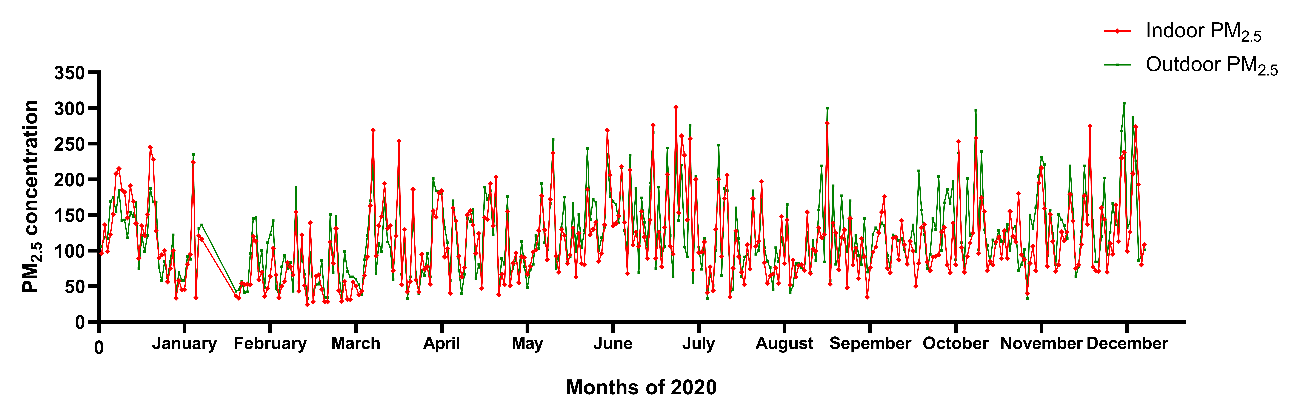


(b)


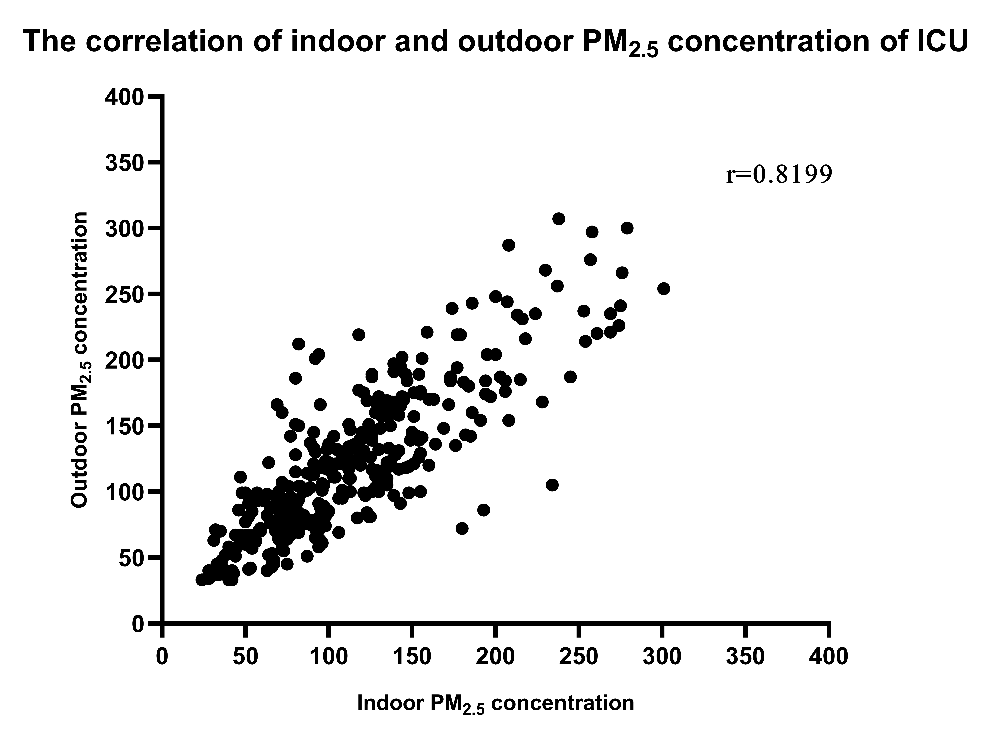


Notes: (a) the value of indoor and outdoor PM_2.5_ concentration in ICU during 2020; (b) spearman correlation of indoor and outdoor PM_2.5_ concentration in ICU during 2020. ICU: intensive care unit.

# Figure S3: Estimated exposure-response curves for short-term exposures to PM_2.5_


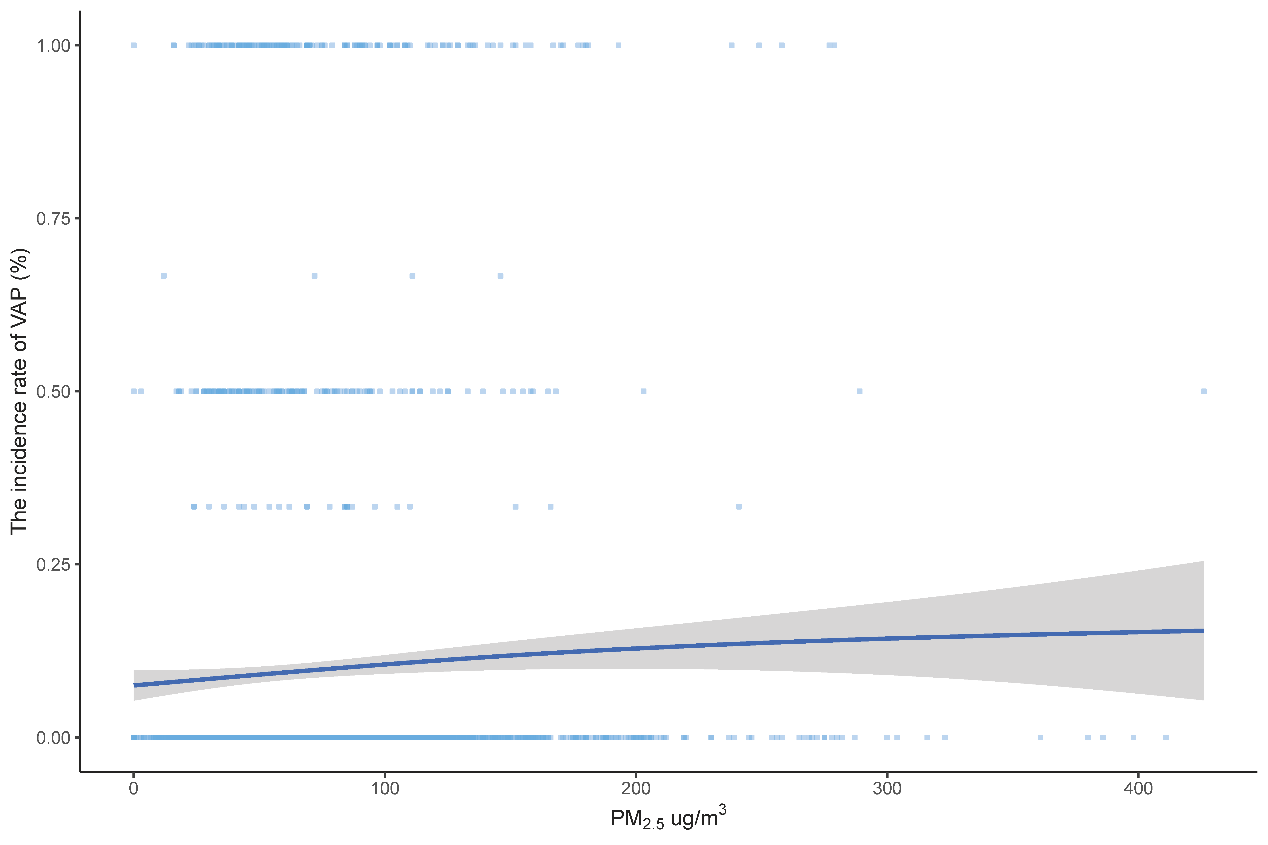


Note: absolute VAP incidence (not RR value of VAP) increased with PM_2.5_ concentration at lag 2-day. VAP: ventilator-associated pneumonia; RR: relative risk.

**References:**

1.Roeleveld PP, Guijt D, Kuijper EJ, Hazekamp MG, de Wilde RB, de Jonge E, (2011) Ventilator-associated pneumonia in children after cardiac surgery in The Netherlands. INTENS CARE MED 37: 1656-1663

2.Ericson JE, McGuire J, Michaels MG, Schwarz A, Frenck R, Deville JG, Agarwal S, Bressler AM, Gao J, Spears T, Benjamin DJ, Smith PB, Bradley JS, (2020) Hospital-acquired Pneumonia and Ventilator-associated Pneumonia in Children: A Prospective Natural History and Case-Control Study. PEDIATR INFECT DIS J 39: 658-664

3.Shaath GA, Jijeh A, Faruqui F, Bullard L, Mehmood A, Kabbani MS, (2014) Ventilator-associated pneumonia in children after cardiac surgery. PEDIATR CARDIOL 35: 627-631

4.Macher J, Gras LGC, Chenouard A, Liet JM, Gaillard LRB, Legrand A, Mahuet J, Launay E, Gournay V, Joram N, (2017) Preoperative Staphylococcus aureus Carriage and Risk of Surgical Site Infection After Cardiac Surgery in Children Younger than 1 year: A Pilot Cohort Study. PEDIATR CARDIOL 38: 176-183

5.Chenouard A, Roze JC, Hanf M, Macher J, Liet JM, Gournay V, Gras-Le GC, Joram N, (2015) Evaluation of the relationship between plasma transfusion and nosocomial infection after cardiac surgery in children younger than 1 year. PEDIATR CRIT CARE ME 16: 139-145

6.Iosifidis E, Pitsava G, Roilides E, (2018) Ventilator-associated pneumonia in neonates and children: a systematic analysis of diagnostic methods and prevention. FUTURE MICROBIOL 13: 1431-1446
